# A Case of IgG4-Related Disease Presenting With Concurrent Uveitis, Posterior Scleritis, Serous Retinal Detachment, and Choroidal Thickening Mimicking Intraocular Lymphoma

**DOI:** 10.7759/cureus.82054

**Published:** 2025-04-11

**Authors:** Hajime Yokota, Seii Kojo, Himeko Kubota, Koichiro Takahashi, Takashi Uno

**Affiliations:** 1 Diagnostic Radiology and Radiation Oncology, Chiba University Graduate School of Medicine, Chiba, JPN; 2 Radiology, Chiba University Hospital, Chiba, JPN; 3 Radiology, Chiba University Graduate School of Medicine, Chiba, JPN; 4 Radiology, Chiba University, Chiba, JPN

**Keywords:** choroidal thickening, igg4-related disease, igg4-related ophthalmic disease, intraocular lymphoma, palatal thickening, posterior scleritis, serous retinal detachment, uveitis

## Abstract

IgG4-related disease (IgG4-RD) is a systemic fibro-inflammatory condition characterized by tumor-like lesions, dense lymphoplasmacytic infiltrates rich in IgG4-positive plasma cells, and often elevated serum IgG4 levels. Ocular involvement is common, typically affecting the lacrimal glands and extraocular muscles. However, intraocular manifestations such as uveitis and scleritis are less frequent. We report the case of a 66-year-old Japanese man presenting with bilateral uveitis, posterior scleritis, serous retinal detachment, and choroidal thickening, initially mimicking intraocular lymphoma. His serum IgG4 and soluble interleukin-2 receptor levels were markedly elevated. Magnetic resonance imaging showed posterior eyeball wall thickening with restricted diffusion and diffuse thickening of the nasal mucosa and hard palate mucosa. Computed tomography revealed an asymptomatic retroperitoneal mass around the aorta. Salivary gland biopsy confirmed dense lymphoplasmacytic infiltration with increased IgG4-positive plasma cells (80/HPF) and an elevated IgG4/IgG ratio (~50%), consistent with IgG4-RD. The patient responded well to oral corticosteroid therapy with improvement in visual acuity. This case highlights the rare but important presentation of intraocular manifestations in IgG4-RD.

## Introduction

IgG4-related disease (IgG4-RD) is recognized as a systemic fibro-inflammatory disorder characterized by the formation of tumor-like lesions in various organs. Histopathologically, it is marked by dense lymphoplasmacytic infiltration rich in IgG4-positive plasma cells, storiform fibrosis, and often obliterative phlebitis [1,2]. Elevated serum IgG4 levels are frequently observed [3]. Initially identified as a cause of autoimmune pancreatitis, the spectrum of IgG4-RD is now known to encompass conditions previously considered distinct entities, affecting nearly every organ system.

Ophthalmic involvement, termed IgG4-related ophthalmic disease (IgG4-ROD), is increasingly recognized. While involvement of lacrimal glands, extraocular muscles, and orbital soft tissues is common [4], eyeball manifestations such as scleritis and uveitis are considered relatively rare [5-7]. Furthermore, the involvement of structures such as the palatine glands, leading to hard palate thickening, is an emerging area of recognition [8]. These less common presentations can pose significant diagnostic challenges, often mimicking other inflammatory or malignant conditions [9,10].

This report presents a case of IgG4-RD in a 66-year-old Japanese man who manifested concurrent bilateral uveitis, posterior scleritis, serous retinal detachment, and choroidal thickening, along with hard palate thickening, initially raising concerns for intraocular lymphoma. We describe the clinical course and diagnostic workup, emphasizing multi-organ assessment, including specific magnetic resonance imaging (MRI) findings and successful management, highlighting the importance of considering IgG4-RD in the differential diagnosis of complex intraocular diseases.

## Case presentation

A 66-year-old Japanese man was referred to our ophthalmology department (Day 0) with suspected bilateral uveitis and posterior scleritis. His ocular symptoms began approximately six months before presentation (around Month -6) with redness and pain in the right eye. His visual acuity at a previous clinic was 0.3 (right eye) and 0.2 (left eye). Treatment with topical steroids led to improvement, with his right visual acuity reaching 0.8 around Month -4. However, inflammation recurred around Month -2, necessitating the resumption of topical steroid therapy. His right visual acuity was 1.0 approximately one month before presentation (Month -1). Four days before the presentation (Day -4), he experienced a sudden decrease in vision in his right eye to 0.09, leading to the referral. He denied any preceding cold symptoms, headache, or current eye pain. His past medical history included surgery for left eyelid xanthelasma approximately five months before presentation.

On initial examination (Day 0), his best-corrected visual acuity was 0.2 in the right eye and 0.5 in the left eye. Slit-lamp examination revealed anterior chamber inflammation, more prominent in the right eye, and bilateral senile cataracts. Fundus examination and optical coherence tomography of the right eye showed serous retinal detachment, retinal pigment epithelium irregularity, and choroidal thickening and elevation. The left eye showed retinal pigment epithelium irregularity without serous retinal detachment. Vitreous opacity was noted bilaterally. B-mode ultrasonography showed subretinal/choroidal lesions, but scleral thickening was unclear.

Initial laboratory (Day 0) tests revealed elevated serum soluble interleukin-2 receptor (sIL-2R) at 1,806 U/mL (reference range: <519 U/mL) and markedly elevated IgG at 3,214 mg/dL (reference range: 870-1,700 mg/dL). Lactate dehydrogenase was within normal limits. IgA was 128 mg/dL, IgM was 52 mg/dL, and IgE was 1,083 IU/mL (elevated).

**Figure 1:**
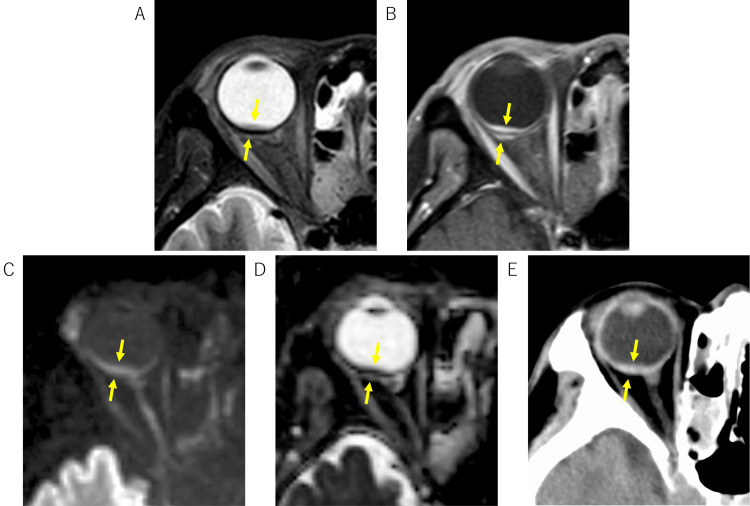
Intraocular findings on MRI and CT. Fat-saturated T2-weighted image shows wall thickening involving the inner and outer aspects of the posterior eyeball wall (A). Post-contrast T1-weighted image demonstrates uniform enhancement of the inner and outer aspects of the thickened wall (B). The lesion exhibits high signal intensity on diffusion-weighted imaging (C) and low apparent diffusion coefficient (D) values. CT shows high density in the corresponding area (E).

**Figure 2:**
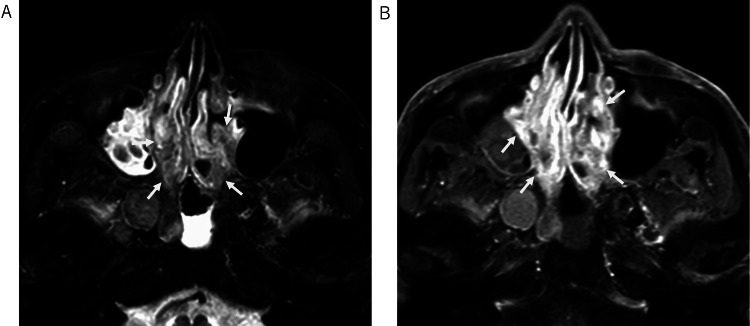
Nasal findings on MRI. Fat-saturated T2-weighted image reveals diffuse and irregular thickening of the nasal mucosa, containing interspersed areas of low signal intensity (A). Extensive enhancement is observed after contrast administration on post-contrast T1-weighted image (B).

**Figure 3:**
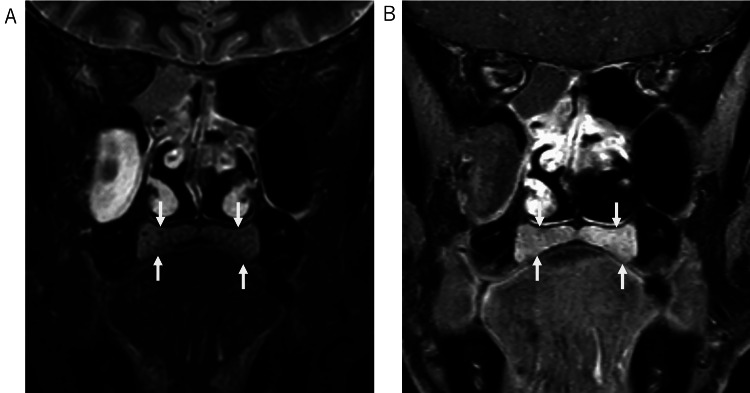
Thickening of the hard palate mucosa. Fat-saturated T2-weighted image (A) and post-contrast fat-saturated T1-weighted image (B) demonstrate diffuse thickening of the hard palate mucosa.

Orbital and brain MRI performed 10 days after presentation (Day +10) demonstrated slight thickening of the posterior wall of the right eyeball on fat-saturated T2-weighted images, restricted diffusion, and contrast enhancement, initially raising suspicion for lymphoma (Figure 1). No abnormalities were found in the optic nerves, extraocular muscles, or infraorbital nerves. The MRI also revealed extensive thickening of the nasal mucosa (Figure 2) and diffuse thickening of the hard palate mucosa (Figure 3). These findings collectively raised suspicion for IgG4-RD.

Based on the MRI findings suggesting IgG4-RD, serum IgG4 level was measured from the blood sample taken at presentation, which returned markedly elevated at 1,890 mg/dL (reference range: 4.8-105 mg/dL).

**Figure 4:**
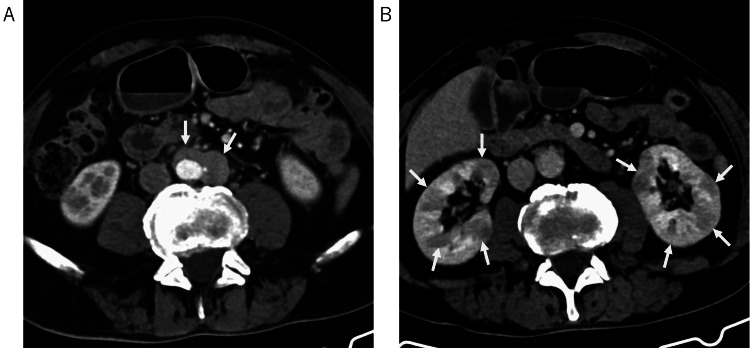
Abdominal findings on CT. Mass-like soft tissue is observed around the aorta (A). Multiple poorly enhancing areas are present in both kidneys (B).

To screen for other organ involvement, a whole-body computed tomography (CT) scan was performed 20 days after presentation (Day +20). This revealed mass-like soft tissue around the abdominal aorta, consistent with retroperitoneal fibrosis/periaortitis, and multifocal areas of reduced enhancement in both kidneys, suggestive of interstitial nephritis (Figure 4). Lymphadenopathy was not observed. The patient was asymptomatic regarding these thoracoabdominal findings.

Although an MRI of the submandibular gland showed no definite abnormalities, ultrasound revealed irregular internal echoes. Therefore, to confirm the diagnosis and rule out malignancy, a submandibular gland biopsy was performed 23 days after presentation (Day +23). The submandibular gland biopsy revealed salivary gland tissue with marked lymphoplasmacytic infiltration, fibrosis, acinar atrophy, approximately 80 IgG4-positive plasma cells per high-power field (HPF), and an IgG4/IgG positive cell ratio estimated at around 50%. These findings were considered suggestive of IgG4-RD [2,10,11].

Based on the multi-organ involvement (eyes, nasal mucosa, salivary glands, hard palate, retroperitoneum, and kidney), elevated serum IgG4 levels, and characteristic histopathological findings, a final diagnosis of IgG4-related disease was established. Oral corticosteroid therapy was initiated, and the patient’s visual acuity showed improvement.

## Discussion

This case illustrates a complex presentation of IgG4-RD characterized by concurrent bilateral uveitis, posterior scleritis, serous retinal detachment, and choroidal thickening. While IgG4-ROD commonly involves the lacrimal glands and extraocular muscles [4], the combination of these specific intraocular findings is relatively rare [5-7]. The initial presentation, particularly the MRI findings of posterior eyeball wall thickening with restricted diffusion, mimicked intraocular malignant lymphoma, posing a significant diagnostic challenge.

The diagnosis of IgG4-RD relies on a combination of clinical, serological, radiological, and histopathological findings [11]. In our patient, suspicion for IgG4-RD arose from the orbital MRI findings, which suggested thickening of the nasal mucosa and hard palate mucosa. Subsequent investigations confirmed multi-organ involvement (retroperitoneum, kidney) through imaging.

The differential diagnosis for posterior scleritis and uveitis is broad, including infectious, autoimmune (e.g., granulomatosis with polyangiitis, sarcoidosis), and neoplastic causes. The constellation of findings in our patient, i.e., posterior scleritis, uveitis, serous retinal detachment, and choroidal thickening, could be seen in conditions such as Vogt-Koyanagi-Harada disease or sympathetic ophthalmia. However, the systemic findings (nasal mucosal thickening, palatal thickening, retroperitoneal fibrosis, and interstitial nephritis) and specific serological/histopathological markers directed the diagnosis toward IgG4-RD.

Furthermore, our patient’s MRI finding of diffuse hard palate thickening is consistent with recent reports on palatine gland involvement in IgG4-RD. Baba et al. found that palatal thickness >8 mm on coronal MRI, along with low T2 signal intensity relative to controls, was characteristic of palatine gland lesion-associated IgG4-RD [8]. This manifestation is often asymptomatic, as in our case, and frequently coexists with other head and neck lesions such as dacryoadenitis. Recognizing this feature can further support the diagnosis and differentiate IgG4-RD from mimics such as lymphoma.

Published case reports of IgG4-ROD presenting with posterior scleritis and/or uveitis are limited but emerging [5-7]. Reports often describe scleritis or uveitis individually or associated with more common manifestations such as dacryoadenitis. Cases combining posterior scleritis, uveitis, serous retinal detachment, and choroidal thickening, as seen here, appear particularly rare.

This case underscores the importance of considering IgG4-RD in patients presenting with atypical or complex intraocular inflammation, even when features suggest malignancy. A comprehensive evaluation including systemic symptom review, multi-organ imaging (paying attention to findings such as palatal thickening), specific serological markers (IgG4, sIL-2R, IgE), and, crucially, tissue biopsy with appropriate immunohistochemistry is necessary for accurate diagnosis and timely initiation of treatment.

## Conclusions

We presented a case of IgG4-RD manifesting with a rare combination of bilateral uveitis, posterior scleritis, serous retinal detachment, and choroidal thickening, which initially mimicked intraocular lymphoma. Diagnosis was achieved through a comprehensive approach integrating clinical findings, multi-organ imaging revealing asymptomatic systemic involvement, including characteristic palatal changes, specific serological elevations (IgG4, sIL-2R), and confirmatory salivary gland histopathology. Prompt diagnosis and initiation of corticosteroid therapy led to favorable outcomes in our patient.
